# Optical Algorithms at Satellite Wavelengths for Total Suspended Matter in Tropical Coastal Waters

**DOI:** 10.3390/s8074165

**Published:** 2008-07-10

**Authors:** Sylvain Ouillon, Pascal Douillet, Anne Petrenko, Jacques Neveux, Cécile Dupouy, Jean-Marie Froidefond, Serge Andréfouët, Alain Muñoz-Caravaca

**Affiliations:** 1 IRD, BP A5, 98848 Nouméa Cedex, New Caledonia; 2 LEGOS/OMP, Université de Toulouse, UMR 5566, 14 avenue Edouard Belin, 31400 Toulouse, France; 3 Laboratoire d'Océanographie Physique et de Biogéochimie, Université Aix Marseille, Centre d'Océanologie de Marseille, Luminy, 13288 Marseille cedex 09, France; 4 Université Pierre et Marie Curie - Paris 6, CNRS, UMR 7621, Avenue Fontaulé, BP44, F-66650 Banyuls-sur-Mer, France; 5 Université de Bordeaux, CNRS, UMR 5805, Bordeaux, F-33000, France; 6 Centro de Estudios Ambientales de Cienfuegos, 5 CP 59 350 Ciudad Nuclear, Cienfuegos, Cuba

**Keywords:** Ocean color, remote sensing, sediment transport, suspended matter, turbidity, New Caledonia, Cuba, Fiji

## Abstract

Is it possible to derive accurately Total Suspended Matter concentration or its proxy, turbidity, from remote sensing data in tropical coastal lagoon waters? To investigate this question, hyperspectral remote sensing reflectance, turbidity and chlorophyll pigment concentration were measured in three coral reef lagoons. The three sites enabled us to get data over very diverse environments: oligotrophic and sediment-poor waters in the southwest lagoon of New Caledonia, eutrophic waters in the Cienfuegos Bay (Cuba), and sediment-rich waters in the Laucala Bay (Fiji). In this paper, optical algorithms for turbidity are presented per site based on 113 stations in New Caledonia, 24 stations in Cuba and 56 stations in Fiji. Empirical algorithms are tested at satellite wavebands useful to coastal applications. Global algorithms are also derived for the merged data set (193 stations). The performances of global and local regression algorithms are compared. The best one-band algorithms on all the measurements are obtained at 681 nm using either a polynomial or a power model. The best two-band algorithms are obtained with R412/R620, R443/R670 and R510/R681. Two three-band algorithms based on Rrs620.Rrs681/Rrs412 and Rrs620.Rrs681/Rrs510 also give fair regression statistics. Finally, we propose a global algorithm based on one or three bands: turbidity is first calculated from Rrs681 and then, if < 1 FTU, it is recalculated using an algorithm based on Rrs620.Rrs681/Rrs412. On our data set, this algorithm is suitable for the 0.2-25 FTU turbidity range and for the three sites sampled (mean bias: 3.6 %, rms: 35%, mean quadratic error: 1.4 FTU). This shows that defining global empirical turbidity algorithms in tropical coastal waters is at reach.

## 1. Introduction

Every year, 20 billion tons of sediments are brought to the oceans by the rivers (Milliman and Syvitski 1992). Among these particles, the finest ones enrich or inhibit the coastal ecosystems, and distribute the fixed or adsorbed pollutants of metal, chemical or organic origin. The coarser particles, mainly sand, constitute the principal source of material fattening the beaches. Coastal morphodynamics results from the balance between land inputs and offshore sediment transport. Studying sediment composition, transport and fate in coastal zones is thus necessary both from the biological point of view (ecosystems' health) and for civil engineering and coastal management. Remote sensing brings efficient tools to monitor sediment transport and analyze the fate and distribution of suspended matter in riverine and coastal waters since it offers a synoptic and instantaneous vision field of the Total Suspended Matter (TSM) concentration (e.g. Holyer 1978; Sydor 1980; Novo et al. 1989; Forget and Ouillon 1998; Ahn et al. 2001, 2006; Doxaran et al. 2002, 2003; Zhang et al. 2003; Ouillon 2003; Wozniak and Stramski 2004; Binding et al. 2005; Bowers and Binding 2006; Teodoro and Veloso-Gomes 2007; Wang et al. 2007). TSM is also referred to as Suspended Particulate Matter (SPM) or Total Suspended Solids (TSS) in the literature. Integrated studies couple numerical simulations of sediment transport and spatial imaging, the images making it possible to gauge and validate the models (Puls et al. 1994; Estournel et al. 1997; Siegel et al. 1999; Ouillon et al. 2004).

However, the fact that no generic algorithm for quantifying TSM in coastal zones has yet been proposed constitutes the principal handicap/barrier to the development of TSM mapping by remote sensing (Acker et al. 2005). The multiplicity of the parameters used to represent directly or indirectly TSM such as its mass concentration (in mg L^-1^), turbidity (in various units, such as Formazin Turbidity Unit or FTU, Nephelometric Turbidity Unit or NTU), light transmission and attenuation, also limits the algorithmic development. Whereas the concentration in chlorophyll-a (chl-a) has been chosen as the main inversion parameter and proxy for phytoplankton pigments, there is not yet a consensus among the specialists in particulate transport to choose a common parameter. In this context, this paper compares several TSM algorithms, in term of turbidity, for three tropical coastal areas. The three sites were selected for the diversity of their respective amounts of suspended matter and chl-a. Then the feasibility to propose a valid single algorithm for different tropical coastal sites is studied.

## 2. Sites and Methods

### 2.1. Study areas and field campaigns

To derive optical TSM algorithms and analyze their variability in tropical coastal waters, physical and optical measurements were performed during several cruises at three sites:

1. the southwest lagoon of New Caledonia, located between (22°10′S, 166°05′E) and (22°40′S, 166°40′E). New Caledonia is an approximately 450 km long × 50 km wide island, surrounded by the second largest coral reef lagoon in the world (22,175 km^2^). The waters in the New Caledonian lagoon are generally oligotrophic, except in the vicinity of Noumea, the main city of New Caledonia, due to anthropogenic disturbances (Torréton et al. 2007; Rochelle-Newall et al. 2008). River inputs are generally extremely low (i.e. < 5 m^3^ s^-1^), except during strong but short rainfall events. The lagoon is semi-enclosed and connected to the Coral Sea through a barrier reef segmented by narrow passes (Jouon et al. 2006). The measurements presented in this paper were performed between June 2002 and February 2006.
2. the Cienfuegos Bay in Cuba, located between (22°12′N, 80°33′W) and (22°04′N, 80°22′W). This bay is almost enclosed, strongly influenced by urban and industrial activities, and connected to the Caribbean Sea by a narrow channel (Alonso-Hernandez et al. 2006, Perez-Santana et al. 2007). The measurements presented in this paper were performed in May 2006. TSM concentrations are, on average, bigger than at the New Caledonia site and smaller than at the Fiji site.
3. the Suva Harbour and Laucala Bay south of Viti Levu, the biggest (10388 km^2^) of the 844 Fiji islands and islets, located between (18°00′S, 178°22′E) and (18°15′S, 178°35′E). The Rewa River, with mean monthly discharges in the range 45-210 m^3^ s^-1^, provides the Laucala Bay with a continuous high amount of suspended particles. Bay muds are about 25-40 m thick at Suva Harbour (Shorten 1993) and the area is also influenced by anthropogenic activities. This lagoon is connected to the Pacific Ocean through a coral barrier reef opened with wide passes (Singh 2007).

While the New Caledonian and Cuban sites are located at tropical latitudes, the Fijian site is located in the intertropical zone. The climate in Fiji is humid and rainfalls are very erosive. Islands from Oceania are known to be a major source of particles to the global ocean (Milliman and Syvitski, 1992). Optical measurements and remote sensing can thus be useful tools to quantify TSM and to study its variability in the context of climate change. The measurements were performed in the Suva Harbour, in the Laucala Bay and in the downstream part of the Rewa estuary (up to ∼10 km from the Rewa River mouth), since the estuary is wide enough (>100m) to map turbidity or TSM from remotely-sensed data. The measurements presented in this paper were performed in April 2003.

Turbidity and chl-a ranges at each site are presented in Table 1. Unfortunately, technical problems did not allow us to measure chl-a properly in the Cienfuegos Bay. However, chl-a was likely higher in the Cienfuegos Bay than in Fiji and New Caledonia.

### 2.2. Instruments and Methods

The measurements consist of remote sensing reflectance (Rrs) spectra deduced from radiance and irradiance measurements that were performed using an Ocean Optics USB2000 radiometer, and turbidity profiles measured with a Seapoint turbidimeter connected to a SBE19 CTD probe. Chl-a concentrations were measured at some stations in New Caledonia and Fiji either by fluorometry or by spectrofluorometry (Neveux and Lantoine 1993) (see Table 1).

The remote sensing reflectance just above the sea surface, Rrs, was measured using the protocol proposed by Mobley (1999), using an optic cable connected to the radiometer. A Gershun tube at the end of the fiber was used to reduce the Field-Of-View (FOV) to an angle of 8°. At each station, measurements were performed for downwelling irradiance *E_d_(0*+*)* using a Spectralon plate, for upwelling radiance *L_u_(0*+*)* with an azimuth viewing direction of 135° from the sun and a zenith angle of 45°, and for sky radiance *L_sky_(0*+*)*. At least 10 measurements of each radiance or irradiance were averaged at every station. Rrs was calculated according to Mobley (1999) by:

(1) $$R_{rs}=\frac{L_{w}\;(0+)}{E_{d}(0+)}=\frac{L_{u}\;(0+)-\rho L_{\text{sky}}(0+)}{E_{d}\;(0+)}$$

where *L_w_(0*+*)* is the water-leaving radiance, and ρ is the proportionality factor that relates the radiance measured when the detector views the sky to the reflected sky radiance measured when the detector views the sea surface. The value of ρ depends on solar zenith angle, on wind speed and on cloud coverage. Under non-cloud conditions and wind speed less than 10 m s^-1^, ρ is not wavelength-dependent, and for wind speed < 5 m s^-1^, *ρ* = 0.028 (Mobley 1999). The protocol is the same and the spectroradiometer of the same kind as the ones used for the measurements performed in the Mediterranean Sea (Ouillon and Petrenko 2005) and in New Caledonia (Ouillon et al. 2004).

Although the Rrs spectra derived from Ocean Optics USB2000 are continuous (2048 channels between 350 and 1000 nm), we only considered, in this paper, the values obtained at a few selected wavelengths which correspond to the wavebands of the major sensors used in coastal oceanography such as MODIS, MERIS, TM, ETM+, SeaWiFS, OCTS. These wavebands (or, to be more precise, the centers of the detected wavebands) are located at 412, 443, 490, 510, 520, 530, 550, 560, 565, 620, 665, 670, 681, 705, 750 and 870 nm.

The Seapoint turbidimeter detects light scattered by particles and uses a 0.88 μm light source wavelength. The sensor was factory adjusted for consistent response to Formazin Turbidity Standard measured in Formazin Turbidity Units (FTU). These sensors, also called nephelometers or Optical Backscatter Sensors (OBS), are known to provide turbidity measurements proportional to sediment concentrations at values less than 10 g L^-1^ (Bunt et al. 1999), the proportionality coefficient varying from a site to another (e.g. Jin et al. 2001). Turbidity values considered in this study were obtained from averaging turbidity profiles from the surface down to 3 m depth. Tests with turbidity averaged over 5 m or 10 m depth (not presented here) generally showed lower correlations with Rrs.

The evaluation of the algorithms is based on criteria used among others by Toole et al. (2000) and Darecki and Stramski (2004). Mean and stdev are defined by:

(2) $$\text{mean}(\text{x})=\bar{x}=\frac{1}{n}\sum_{i=1}^{n}x_{i}\;$$

(3) $$\text{stdev}(\text{x})={\left[\frac{1}{n-1}\sum_{\text{i}=1}^{n}{\left(x_{i}-\bar{x}\right)}^{2}\right]}^{1/2}$$

From these equations, the mean normalized bias (MNB) and the normalized root mean square (rms) error, in percent, are calculated following:

(4) $$\text{MNB}=\text{mean}\left(\frac{y_{\text{alg}}-y_{\text{obs}}}{y_{\text{obs}}}\right).100\;$$

(5) $$\text{rms}=\text{stdev}\left(\frac{y_{\text{alg}}-y_{\text{obs}}}{y_{\text{obs}}}\right).100$$

where *y_alg_* is a variable obtained from an algorithm and *y_obs_* is its value measured *in situ*. MNB is an indicator of systematic error and rms an indicator of random error. Slope, intercept and the squared correlation coefficient (coefficient of determination r^2^) were also calculated for the linear regression of turbidity estimations (from various algorithms) versus turbidity measurements.

To be validated, algorithms must be tested over independent data set. Their application to the training data set does not allow their validation, but provides statistics such as regression slope and intercept, r^2^ and rms error. These statistics constitute a numerical index of model performance which can be compared to those of other models (see e.g. O'Reilly et al. 1998); they are given in this paper for every regression relationship.

## 3. Local algorithms for turbidity

### 3.1. New Caledonia

**Figure 1.**
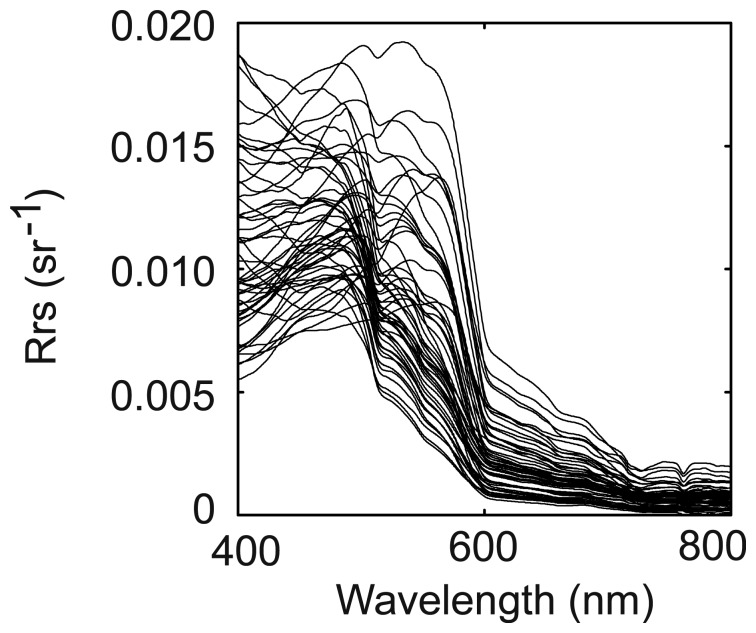
Examples of Rrs spectra recorded in New Caledonia (55 out of 113), 2002-2006.

Reflectance spectra recorded in New Caledonia exhibit the shapes shown in Fig. 1 (for clarity, only 55 spectra out of the 113 used in the present study are shown).

So as to propose one-band algorithms, we calculated coefficient of determination between Rrs(λ) and turbidity averaged over 3 m depth below the sea surface, at every wavelength and for different kinds of regression relationships (linear, polynomial, power, exponential, logarithmic). Rrs and turbidity are highly correlated (r^2^>0.9) with polynoms of degree 3 between 565 and 705 nm and with polynoms or linear relationships between 620 and 705 nm. The best power relationship is obtained at 565 nm; it is well adapted for low turbidity values (≤ 3 FTU) but is less suitable at higher turbidity. An exponential regression relationship is suitable at wavelengths between 550 and 565 nm.

**Figure 2.**
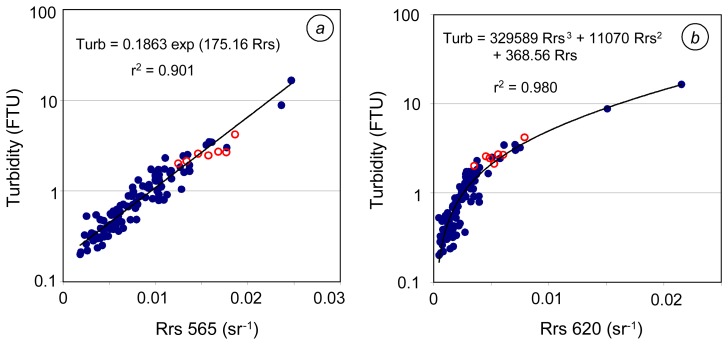
Best-fit single-band algorithms for turbidity in New Caledonia (N=113). 7 stations out of the 113 stations are distinguished with empty red marks; they correspond to the head of bays. **(a)** regression relationship at 565 nm. **(b)** regression relationship at 620 nm. Note that r^2^ values given in figures 2, and after, were calculated between turbidity and reflectance or reflectance ratio, not between measured and modeled turbidity as in Table 2.

Amongst the one-band algorithms for turbidity, the best-fits were established with an exponential relationship at 565 nm (Fig. 2a) and with a cubic polynomial relationship at 620 nm (Fig. 2b). The mean bias and rms error are lower for the regression at 565 nm than for the one at 620 (see Table 2), and the signal amplitude is higher with Rrs565 [short notation hereafter used for Rrs at 565nm]. The Rrs620-based algorithm induces a lower mean quadratic error; it is thus more suitable at high turbidity values than the Rrs565-based algorithm. Consequently, for New Caledonia, the 565 nm exponential relationship seems more suitable to low turbid waters (in dry season or fair weather condition, i.e. in general conditions when satellite data are available with few clouds and no foam), and the 620 nm cubic polynomial algorithm can be preferred for the highest turbid waters (i.e. just after high discharge episodes or high resuspension events). This result is in agreement with a previous study performed on a sub-data set which enabled to draw a map of turbidity from ETM+ band 2 (centered at 565 nm) data in support of calibration of a numerical model for fine suspended sediment transport during the dry season (Ouillon et al. 2004). The highest sensitivity of reflectance for low TSM concentration was already reported to be at shorter wavelengths (Holyer 1978).

Amongst the 113 stations of New Caledonia, 7 are located in bays and are subject to strong anthropogenic influences (Fig. 2). As these stations exhibit high TSM and high amount of organic matter, we calculated the statistics of the regression relationships both for the whole data set (N=113) and excluding the 7 peculiar stations (N=106) (see Table 2). The results show that the 7 “high organic matter” stations (due to urban and domestic waste waters) do not strongly affect the statistical performance of the global one-band TSM algorithms, and thus that these stations are not outliers.

**Figure 3.**
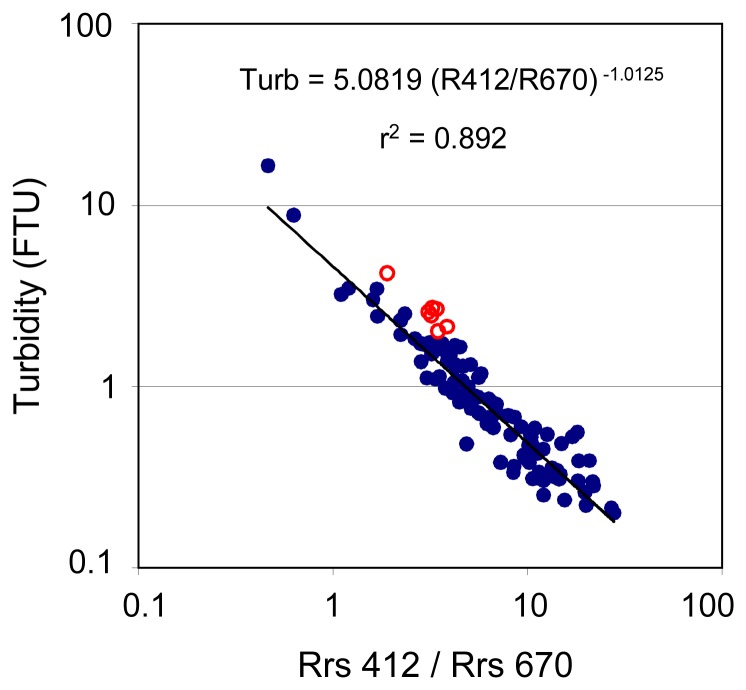
Best-fit two-band algorithm for turbidity in New Caledonia (N=113). 7 stations at the head of bays are distinguished with empty red marks.

Apart from one-band wavelength algorithms, we examined the correlation between reflectance ratios at the central wavelengths of generic bands and turbidity. The best correlation was obtained using the ratio Rrs412/Rrs670 [also hereafter noted R412/R670 since the reflectance ratio is the same as the ratio of remote sensing reflectances] (Fig. 3); close performances were obtained using ratios R412/R620 and R443/R670.

What is the difference between the one- and two-band algorithms? In the R412/R670 ratio-turbidity relationship, the 7 stations at the head of bays are slightly out of the main trend (Fig 3), while they totally fit in the Rrs565- or Rrs620-turbidity relationships (Fig. 2). Also, comparing the results with all the data (case *a*) and the one without the 7 specific stations (case *b*), one notices that the rms error increases from case *b* to case *a* using R412/R670, while it remains quite the same for both cases using Rrs565 (Table 2). Indeed, the ratio R412/R670 is more sensitive to the content in organic matter than single-wavelength reflectance at 565 or 620 nm. In these bays, the residence time is the highest in the southwest lagoon of New Caledonia (Jouon et al. 2006) and coastal waters are rich in organic matter (Torréton et al. 2007). The differences between performances of algorithm in cases *a* and *b* being higher when using the ratios R412/R620 and R443/R670, we only presented the two-band algorithm based on R412/R670.

### 3.2 Cuba

**Figure 4.**
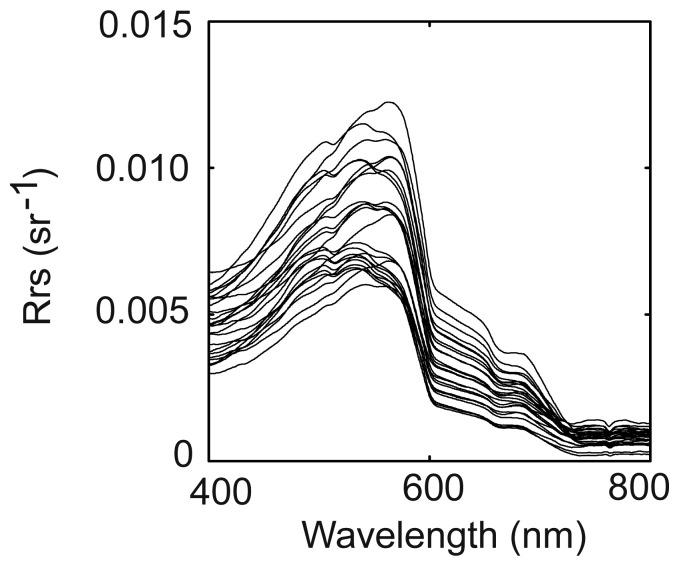
Rrs spectra recorded in Cienfuegos Bay, Cuba, May 2006 (N=24).

**Figure 5.**
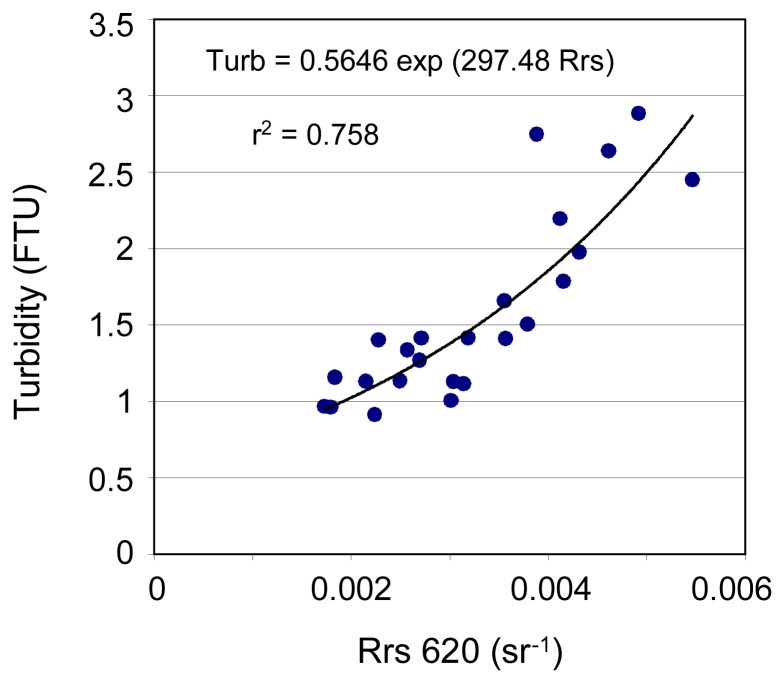
Best-fit single-band algorithm for turbidity in Cienfuegos Bay, Cuba (N=24).

The Rrs spectra collected in May 2006 are presented in Fig. 4. The best one-band algorithm is obtained between Rrs620 and turbidity averaged over 3 m depth (mean bias: 1.4%, rms error: 17.3%, see Fig. 5 and Table 2). However, caution must be taken because these results are obtained with a relatively small data set (24 stations).

Slightly lower statistical performances are obtained between Rrs681 and turbidity using a power model (see Table 2). And still lower statistical performances are obtained with Rrs705. The two-band algorithms show poor statistical performances in Cuba, where chl-a concentrations are likely the highest of the three sites, on average.

### 3.3 Fiji

**Figure 6.**
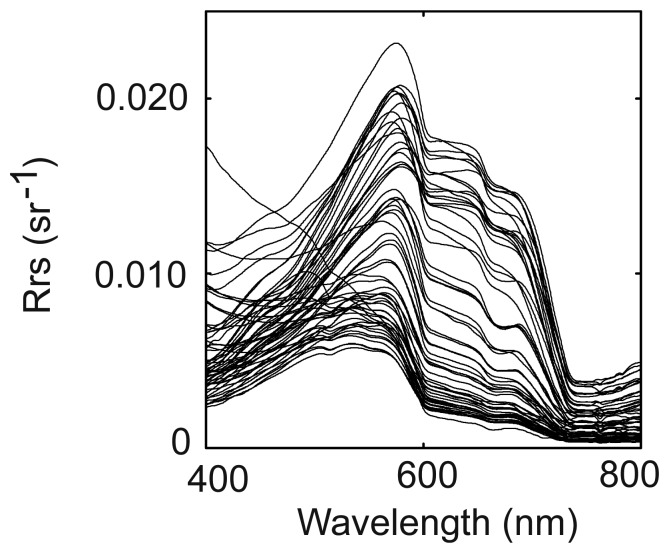
Rrs spectra recorded in Laucala Bay and in the Rewa estuary, Fiji, April 2003.

**Figure 7.**
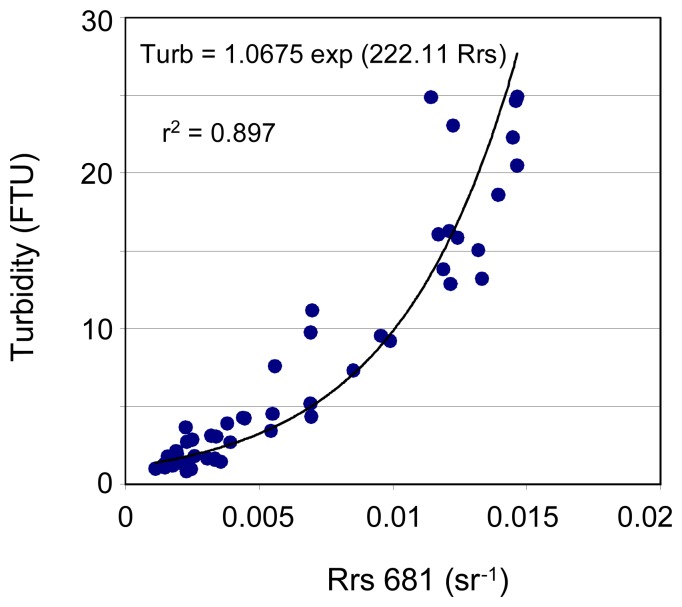
Example of single-band algorithm for turbidity in Laucala Bay, Suva Harbour and Rewa estuary, Fiji, based on Rrs at 681 nm.

**Figure 8.**
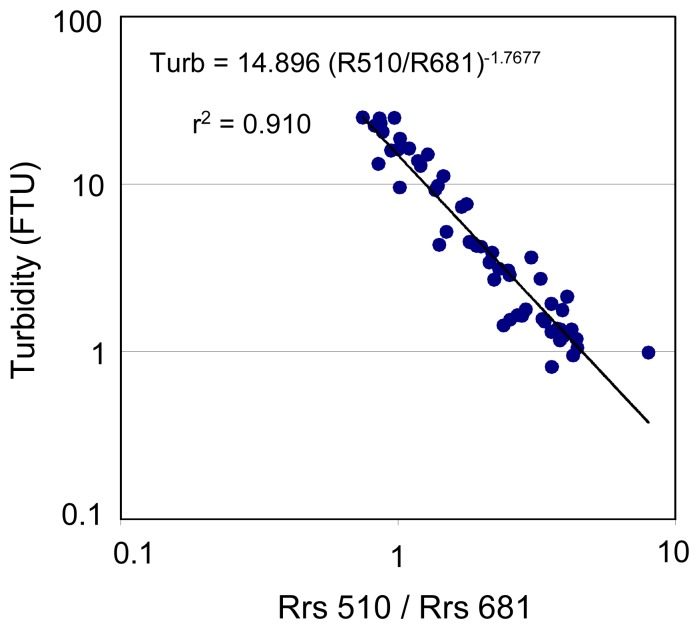
Best-fit two-band algorithm for turbidity in Laucala Bay, Suva Harbour and Rewa estuary, Fiji.

Rrs measurements performed in Fiji are presented in Fig. 6. The best-fit single-band algorithms for turbidity are found based on Rrs620 and Rrs681 (Fig. 7), while the best-fit two-band algorithm is based on R510/R681 following a power model (Fig. 8) (see also Table 2). Turbidity calculated using these relationships shows a mean normalized bias of less than 6% and a rms error of ∼35% compared to the measured values (see Table 2). However, the two-band algorithm provides a lower mean quadratic error than the one-band algorithms.

## 4. Towards a global algorithm in tropical coastal waters

There is a strong demand from potential users to dispose of global TSM algorithms (Acker et al. 2005), but the first question remains: is it possible? To examine this question and bring some reply elements regarding tropical coastal waters, we merged the 3 data sets and examined the feasibility of building a global Rrs-turbidity algorithm.

### 4.1 Correlation between surface turbidity and Rrs(λ), one-band algorithms

Between 400 and 500 nm, the relationship is not univocal between Rrs and turbidity. In New Caledonia and Fiji, the smallest and highest reflectance values correspond to low turbidities while high turbidities are accompanied with intermediate Rrs. In Cuba, the higher turbidity, the larger Rrs in the range 400-550 nm.

Beyond 500 nm in Fiji and Cuba, 550 nm in NC, reflectance increases with increasing turbidity. Points are very dispersed at 510 and 520 nm and the relationship only becomes significant at 550 nm (r^2^ > 0.5 for the most adapted law, generally polynomial - see e.g. Fig 2a). In New Caledonia, r^2^ increases very quickly with increasing wavelength (0.78 at 550 nm, 0.90 at 560 nm, 0.923 at 565 nm), and is very high between 620 nm (0.976) and 681 nm (0.968). However differences between Rrs values diminish with wavelengths beyond 565 nm. At 620 nm and above, two points corresponding to two very turbid stations (at a river mouth) are clearly distinguished and explain the good performances of the correlations (see Fig. 2b). The correlation is as good at 705 nm as at 681 nm, then it quickly degrades with increasing wavelength (r^2^ = 0.74 at 750 nm and 0.47 at 870 nm). In Cuba, the best correlations are obtained between 620 and 681 nm (r^2^ ranging between 0.72 and 0.78), r^2^ is worth 0.55 at 560 nm and 0.61 at 705 nm. However these results are not completely satisfactory because more than half of the stations show close turbidities (between 1 and 1.5 FTU) and very dispersed Rrs values. Beyond 750 nm, points are totally dispersed. In Fiji, the turbidity-Rrs relationship presents a strong dispersion up to 600 nm, in particular at high turbidities (> 5 FTU), and beyond 705 nm. The best correlations are obtained between 620 and 681 nm.

When considering all the stations without distinguishing the sites, one checks that the correlation between turbidity and reflectance is significant only beyond 550 nm, and is the best between 620 nm and 705 nm. This result is consistent with several papers in which reflectance in the red band (600-700 nm, either broadband or at the center wavelength) provides the best one-band relationships with TSM (e.g. Ahn et al. 2001, 2006; Hu et al 2004). The performances of the regression relationships between 620 nm and 705 nm are equivalent; however paying a detailed attention to the curves points to differences which can guide the choice of a law rather than another. Rrs620 variations are very small at the strongest turbidities (ranging between 20 and 25 FTU, measured in Fiji), whereas Rrs705 is still sensibly increasing with increasing turbidity in this range. Nonetheless, the disadvantage of high wavelengths is that the range of Rrs values decreases with wavelength in the red and infrared, as already shown by Novo et al. (1991).

At 620, 665, 670, 681 and 705 nm, linear relationships between Rrs and turbidity averaged over 3 m depth provide correlation coefficients close to the ones for power functions (where Turb=*a* Rrs*^b^*). The power coefficient *b* decreases with increasing wavelength (1.29 at 620 nm, 1.25 at 685 nm, 1.19 at 705 nm). Han (1997) already showed that the linearity in the TSM-reflectance relationship increases with increasing wavelength between 400 and 900 nm. Overall, the power coefficient is relatively close to 1, thus explaining the similar performance of both kinds of relationships: linear and power.

At 620 nm, the best statistical performances are obtained with a polynom of degree 3 which makes it possible to take into account the damping of Rrs for strong turbidities. At 681 nm, the average relative error is higher and the quadratic error is lower with a polynomial than with a power law. The power-law model is less powerful for strong turbidities but more adapted to low turbidities.

**Figure 9.**
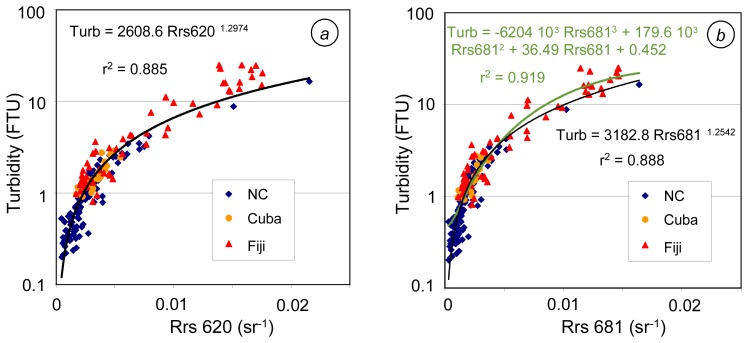
Single-band algorithms for turbidity in tropical waters (N=193: 113 stations in New Caledonia, 24 in Cuba and 56 in Fiji). **(a)** power law at 620 nm. **(b)** power law and polynomial relationship at 681 nm.

Finally, the best single-band algorithms are obtained between turbidity averaged over 3 m depth and Rrs at 620 or 681 nm. The relationships with Rrs681 give slightly better results than those with Rrs620 (Fig. 9). When the power relationship at 681 nm is used to calculate turbidity from Rrs, the mean normalized bias is 7.6% (see Table 3 and Fig. 9a), the mean bias being +17.5%, -1.4% and -8.6% over the New Caledonian, Cuban and Fijian data, respectively; the mean quadratic errors are lower than 1 FTU (0.75 FTU in New Caledonia, 0.36 in Cuba), except in Fiji (3.3 FTU) (see Table 3 and Fig. 9b). The polynomial relationship at 681 nm gives better turbidity estimates in Fiji (mean quadratic error of 2.4 FTU instead of 3.3, and rms error of -3.3%). The power law at 681 nm will preferably be applied to waters with a turbidity < 1 FTU, and will advantageously be replaced by a polynomial law for more turbid waters.

### 4.2 Two-band algorithms

Concerning global two-band algorithms, we examined the correlations of all reflectance ratios (hereafter N/D for numerator/denominator) with turbidity. The best reflectance ratios to derive turbidity (Turb in following formula) were obtained with N = Rrs412, Rrs443 and Rrs510, and D = Rrs620, Rrs670 and Rrs681. Others do not fit, such as R510/R550, because the *b* exponent in the Turb=a (R510/R550)^b^ relationship varies from a site to another (e.g. *b*=-2.85 in New Caledonia, *b*=-6.6 in Fiji), and because R510/R550 shows a better correlation with turbidity averaged over 10 m depth than over 3 m depth with our data set.

**Figure 10.**
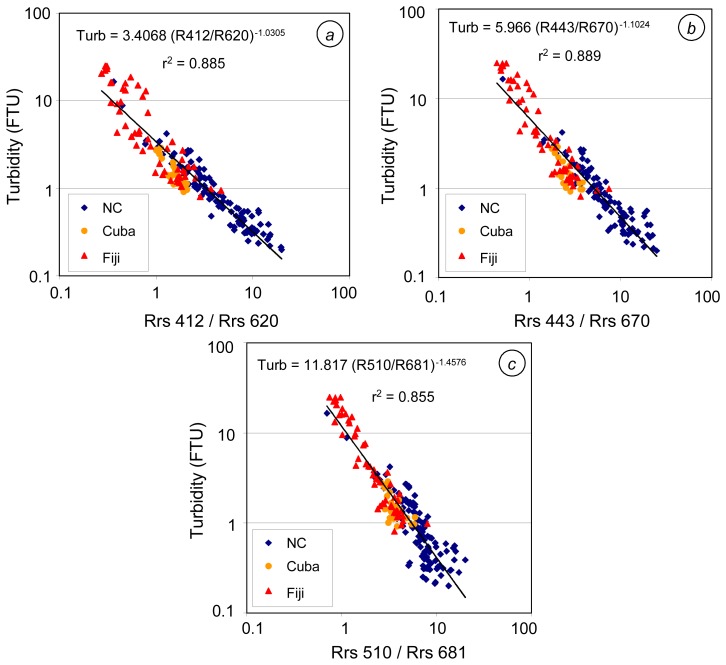
Best-fit band ratio algorithms for turbidity in tropical waters (N=193: 113 stations in New Caledonia, 24 in Cuba and 56 in Fiji) **(a)** using R412/R460. **(b)** using R443/R670. **(c)** using R510/R681.

The relationships derived using these nine ratios were carefully compared. The best statistical performances were obtained with R412/R620, R443/R670, and R510/R681 (Fig. 10, Table 3). The relationships with N=Rrs412 provide good estimates of low turbidity, but very dispersed estimates for turbidity > 2 FTU; D=Rrs620 giving slightly better estimates than D=Rrs670 or D=Rrs681 (Fig. 10a). With N=Rrs510, the higher turbidity estimates are less dispersed than the low turbidity (< 1 FTU) ones; the best relationship being obtained with D=Rrs681 (Fig. 10c). The ratio R443/R670 constitutes a compromise between those two ratios since estimated- versus measured-turbidity globally shows a smaller dispersion than R412/R620 at high turbidity, and than R510/R681 at low turbidity (Fig. 10b). The statistical performances of R443/R670 and R412/R620 are similar: acceptable in New Caledonia (R443/R670 is better since the mean quadratic error is reduced), but bad at Cuba and Fiji stations (high MNB and high quadratic errors, see Table 3). The relationship based on R510/R681 is better in Cuba and Fiji, but is not as precise as R443/R670 in New Caledonia. Green/red band ratios were also found to be best correlated with concentration of mineral suspended solids in coastal waters (Topliss et al. 1990; Wernand et al. 1998; Bowers and Binding 2006); a blue/red ratio was also proposed by Wernand et al. (1998).

### 4.3 Three-band algorithms

**Figure 11.**
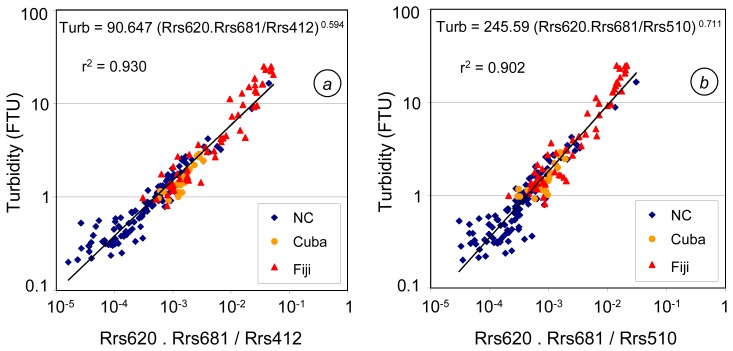
Best-fit three band algorithms for turbidity in tropical waters (N=193: 113 stations in New Caledonia, 24 in Cuba and 56 in Fiji) **(a)** using Rrs620.Rrs681/Rrs412.

As the most performing turbidity algorithms previously presented are based on Rrs620 and Rrs681 (one-band), or R412/R620, R443/R670 and R510/R681 (two-band), we tested algorithms involving three bands in a combination of these channels and ratios. Algorithms based on R670/R443 (either multiplied by Rrs620 or Rrs681) show the lesser fit with turbidity. The two best-fit algorithms are based on Rrs620.Rrs681/Rrs412 (algorithm 6, see Fig. 11a, Table 3) and Rrs620.Rrs681/Rrs510 (algorithm 7, see Fig. 11b, Table 3). Algorithm 6 shows better performances with the New Caledonia stations, while algorithm 7 is more suitable for application in Cuba and in Fiji. Amongst all the algorithms previously tested, algorithm 6 shows the best performance for New Caledonia (lower MNB, 1.9%, lower mean quadratic error, 0.352, see Table 3), and algorithm 7 is one of the best algorithms for Cuba, with the one-band Rrs681-based algorithms.

### 4.4 Proposal for a global algorithm with threshold

The performances of the different global algorithms (Table 3) indicate that none is the best one either globally or for each of the three sites separately. The lower mean bias and rms values were obtained with algorithm 6 because it was the best-performing algorithm for New Caledonia (113 stations out of 193). However, its average statistical performances in the more turbid waters of Cuba and Fiji imply a relatively high mean quadratic error (2 FTU). The one-band polynomial relationship based on Rrs681 is shown as the best algorithm for both Cuba and Fiji (low MNBs, low mean quadratic errors). Nonetheless, this algorithm failed to properly estimate turbidity < 1 FTU (see its performances in New Caledonia, Table 3, and Fig. 9b).

In an attempt to build a better global algorithm, we thus propose to merge the two best-performing relationships in one formulation, following:

(a) turbidity is calculated using algorithm 2:
  (6) $$\text{Turb}=-6204217\;{\left(\text{Rrs}681\right)}^{3}+179652\;{\left(\text{Rrs}681\right)}^{2}+36.49\;\text{Rrs}681+0.452$$
(b) if the resulting turbidity < 1 FTU, its calculation is replaced by algorithm 6:
  (7) $$\text{Turb}=90.647\;{\left(\text{Rrs}620.\text{Rrs}681/\text{Rrs}412\right)}^{0.594}$$

**Figure 12.**
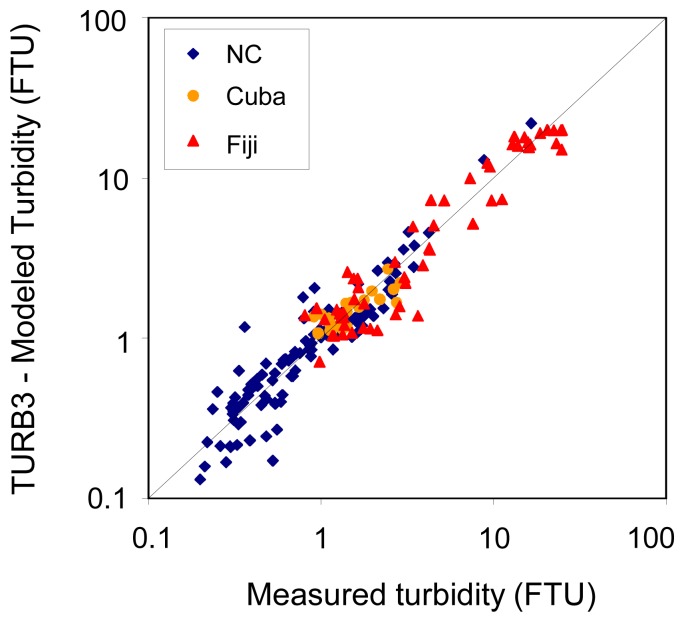
Comparison of measured turbidity (averaged over 3 m below the sea surface) and turbidity calculated from Rrs412, Rrs620 and Rrs681 using TURB3 algorithm (N=193).

As this algorithm is based on three wavelengths (412, 620 and 681 nm, available e.g. with MERIS), we propose to call it hereafter TURB3. Its statistical performances show its ability to estimate turbidity with a mean bias of 3.6%, a rms error of 35% and a mean quadratic error of 1.4 FTU (Table 3, Fig. 12). Algorithm 6 induces lower rms errors both globally and in New Caledonia, but fails to estimate higher turbidity (as indicated by higher rms errors in Cuba and Fiji). TURB3 enables to significantly reduce the mean quadratic error with the Fiji data (2.4 FTU against 3.7 with algorithm 6).

## 5. Discussion and Conclusion

Although the reflectance-TSM relationship is known to vary with the change in particle properties such as grain size, composition and refraction index (Wozniak and Stramski 2004; Binding et al. 2005), results presented in this paper show it is possible to quantify turbidity from remote sensing reflectance in coastal tropical waters.

The debate is still open on defining the good protocol for measuring TSM in-water in order to produce a quantity that is comparable to a remotely-sensed data product (Acker 2006). Several proxies can be used for TSM concentration. Amongst them, turbidity is a simple parameter, both because it can be easily measured and because it is already an optical parameter closely related to the backscatter properties of total suspended matter. Turbidity and TSM were proved to be closely related when TSM is mainly composed of fine particles, i.e. silt and clays (see a review in Ouillon et al. 2004, and analysis in New Caledonia waters in Jouon et al. submitted). It is suggested that the comparison of statistical performances of optical algorithms for other TSM-related bulk parameters, like concentration (in mg/L), turbidity expressed in NTU, light transmission and attenuation, should also be analyzed and presented in future papers.

When considering several sites or different ranges of turbidity within a site, one-band algorithms can be proposed at a wavelength increasing with turbidity, and thus adapted for a given turbidity range. In New Caledonia (average turbidity: 1.23 FTU), Rrs565 is shown to be of preferable use over low turbid waters and Rrs620 more adapted for the highest encountered turbidities. The best-fit one-band algorithms in Cuba and Fiji were based on Rrs620 and Rrs681 (average turbidity: 1.57 and 7 FTU, respectively). It was already shown in the literature that, when turbidity or TSM grows, Rrs increases firstly at green wavelengths between 500 and 600 nm, secondly at higher wavelengths between 600 and 700 nm, and thirdly in the near-infrared around 800 nm (see e.g. reflectance spectra at the Rhône River mouth in Forget and Ouillon 1998, and in the Gironde and Loire estuaries in Doxaran et al. 2003). It is a bit surprising to find the best performing one-band algorithm at 681 nm since this wavelength corresponds to the peak in chlorophyll-fluorescence and is consequently not used *a priori* in TSM algorithms.

In tropical coastal waters, blue/red (412/620, 443/670) and green/red (510/681) ratios are best suitable for two-band turbidity algorithms, while blue and red (412, 620, 681 nm) or green and red wavelengths (510, 620, 681 nm) give the best performances with three-band algorithms.

Algorithms based on reflectance ratios are attractive since there exists several protocols for above-water Rrs measurements (Mueller et al. 2003) and, when shifting from a couple instrument/protocol to another, differences between measurements can be greater amongst absolute Rrs values than amongst reflectance ratios (see e.g. a comparison between SIMBAD and Ocean Optics Rrs measurements at four wavelengths in Ouillon and Petrenko 2005). For application to satellite data, reflectance ratios are also less sensitive to uncertainty in atmospheric correction than absolute reflectance values (Bowers and Binding 2006). However, using reflectance ratios involves a strong disadvantage: algorithms based on reflectance ratios such as R412/R670 or R510/R550 are likely more sensitive to the content of organic matter than single-band algorithms at red or near-infrared wavelengths. Other ratios proposed in the literature involve red and near-infrared bands (Moore et al. 1999; Doxaran et al. 2003), but they are expected to be particularly adapted to waters that are more turbid (with TSM of several tens or hundreds of mg L^-1^) than in coral reef lagoons.

Global algorithms were tentatively derived from the entire data set and were shown to estimate turbidity with a rms error of 30-35%. When we used the derived algorithms to model turbidity, rms errors between modelled and measured turbidity varied from a site to another : ∼38% in New Caledonia, 20% in Cuba and 33% in Fiji using TURB3 algorithm, and ∼28% in New Caledonia, 21% in Cuba and 34% in Fiji using algorithm 6.

Instead of proposing algorithms ≪ per turbidity range ≫, like TURB3 which has two relationships below and above 1 FTU as estimated by a first equation, a test could be done to determine the coastal water type of each marine station and then apply a specific algorithm per water type (see e.g. a coastal water classification and its application by Lahet et al. 2001a, 2001b). Better performances should tentatively be looked for in this way. However, the overall performance of the algorithms presented here is encouraging to carry on and complete such studies. Indeed, the algorithms should be improved and investigations on the different parameters used as proxies for TSM should continue. In tropical waters that are generally not very turbid, the performance of TSM algorithms may also likely increase when the inversion of the satellite data will take into account the stratification in TSM (or turbidity) rather than being based on surface values or values averaged in a surface layer (Ouillon 2003). The approach of this paper, testing optical TSM algorithms at selected wavelengths, must be enlarged and adapted for each satellite sensor, considering the spectral sensitivity of each bands, and algorithms must be validated.

## Figures and Tables

**Table 1.** Turbidity range, averaged over 3m depth below the surface (in FTU) and standard deviation of averaged values (in %), and chlorophyll-a concentration at 1.5 m depth, per site during the field campaigns considered.

	Turbidity (FTU)					Chlorophyll a (mg m^-3^)				

	N	Average (0-3 m)	SD (%)	Min	Max	N	Average (1.5 m)	SD (%)	Min	Max
New Caledonia	113	1.23	147.7	0.20	16.50	84	0.750	0.91	0.08	5.78
Cuba	24	1.57	38.4	0.91	2.88	0				
Fiji	56	7.08	106.0	0.81	24.90	49	2.25	1.47	0.55	9.14
ALL	193	2.96	168.8	0.20	24.90					

**Table 2.** Statistical performance of local turbidity algorithms for New Caledonia (*a*: test over 113 stations; *b*: test over 106 stations, all except anthropogenic bays), Cienfuegos lagoon in Cuba (N=24), and Suva Harbour/Laucala bay in Fiji (N=56). The parameters are obtained between modelled and measured turbidity (averaged over 3m depth below the surface).

Site	Local algorithm	MNB (%)	rms (%)	Mean quadr. error	Slope	Intercept	r^2^
NC (*a*)	Turb=0.1863 exp(175.1 Rrs565)	3.11	25.51	0.481	0.960	0.040	0.931
NC (*b*)		2.49	25.52	0.460	0.943	0.030	0.937
NC (*a*)	Turb=329589 Rrs620^3^+ 11070 Rrs620^2^ + 368.56 Rrs620	17.9	44.48	0.290	0.997	0.067	0.976
NC (*b*)		19.6	45.32	0.286	1.001	0.074	0.978
NC (*a*)	Turb=5.0819 (R412/R670) ^-1.0125^	3.71	28.24	0.629	0.733	0.249	0.919
NC (*b*)		6.47	26.93	0.588	0.752	0.265	0.930

Cuba	Turb=0.565 exp(297.5 Rrs620)	1.43	17.26	0.302	0.717	0.419	0.741
Cuba	Turb=0.552 exp(441.4 Rrs681)	1.83	19.83	0.336	0.632	0.543	0.681

Fiji	Turb=0.928 exp(191.3 Rrs620)	5.49	34.39	3.199	0.923	0.431	0.824
Fiji	Turb=1.068 exp(222.1 Rrs681)	5.90	35.41	2.892	1.019	-0.113	0.871
Fiji	Turb=14.896 (R510/R681) ^-1.768^	5.44	35.90	2.399	0.828	0.743	0.904

**Table 3.** Statistical performance of global turbidity algorithms for New Caledonia, Cienfuegos lagoon in Cuba, and Suva Harbour/Laucala bay in Fiji (N=193). The parameters are obtained between modelled and measured turbidity (averaged over 3m depth below the surface).

	Global statistics						New Caledonia			Cuba			Fiji		

Algorithm	MNB (%)	rms (%)	Mean quadr error	slope	Intercept	r^2^	MNB (%)	rms (%)	Mean quadr error	MNB (%)	rms (%)	Mean quadr error	MNB (%)	rms (%)	Mean quadr error
(1) Turb=3183 (Rrs681) ^1.254^	7.6	45.2	1.832	0.716	0.478	0.913	17.50	50.4	0.411	-1.4	24.0	0.350	-8.7	34.9	3.342
(2) Turb=-6204217(Rrs681)^3^ +179652 (Rrs681)^2^ +36.49 Rrs681 + 0.452	18.5	49.7	1.424	0.919	0.242	0.919	34.9	53.9	0.748	-7.9	19.5	0.365	-3.3	34.7	2.409
(3) Turb=3.407 (R412/R620) ^-1.031^	7.1	39.0	2.832	0.499	0.907	0.812	-3.5	23.7	0.739	53.4	27.6	0.758	8.4	51.7	5.128
(4) Turb=5.966 (R443/R670) ^-1.102^	7.0	39.4	2.453	0.565	0.763	0.887	-3.5	28.7	0.527	51.9	36.9	0.770	8.9	45.4	4.463
(5) Turb=11.817 (R510/R681) ^-1.458^	9.6	49.1	1.995	0.684	0.521	0.899	6.4	53.4	0.583	30.0	39.7	0.588	7.1	41.6	3.589
(6) Turb=90.647 (Rrs620.Rrs681/Rrs412) ^0.594^	4.4	30.1	2.030	0.654	0.619	0.917	1.9	28.2	0.352	25.2	20.7	0.406	0.4	33.8	3.725
(7) Turb=245.59 (Rrs620.Rrs681/Rrs510) ^0.711^	6.5	40.7	1.915	0.701	0.522	0.903	9.1	45.2	0.573	12.2	24.6	0.333	-1.4	35.8	3.454
(8) TURB3: (2) and if Turb < 1FTU, (6)	3.6	35.0	1.416	0.923	0.201	0.920	5.0	38.3	0.731	1.6	19.8	0.350	1.5	33.4	2.405
